# Effects of Different Cooking Methods on Phenol Content and Antioxidant Activity in Sprouted Peanut

**DOI:** 10.3390/molecules28124684

**Published:** 2023-06-10

**Authors:** Liangchen Zhang, Haolin Qu, Mengxi Xie, Taiyuan Shi, Puxiang Shi, Miao Yu

**Affiliations:** 1 Institute of Food and Processing, Liaoning Academy of Agricultural Sciences, Shenyang 110161, China; nap831214@126.com (L.Z.);; 2 Food Science College, Shenyang Agricultural Unversity, Shenyang 110866, China; 3 Institute of Sandy Land Management and Utilization of Liaoning, Fuxin 123000, China

**Keywords:** peanut sprout, cooking method, phenolic compound, antioxidant activity

## Abstract

Peanut sprout is a high-quality healthy food, which not only has beneficial effects, but also a higher phenol content than peanut seed. In this study, peanut sprout was treated with five cooking methods, namely boiling, steaming, microwave heating, roasting, and deep-frying, and the phenol content, monomeric phenol composition, and antioxidant activity were determined. The results showed that, compared with unripened peanut sprout, the total phenol content (TPC) and total flavonoid content (TFC) decreased significantly after the five ripening processes, and the highest retention of phenols and flavonoids was associated with microwave heating (82.05% for TPC; 85.35% for TFC). Compared with unripened peanut sprout, the monomeric phenol composition in germinated peanut was variable after heat processing. After microwave heating, except for a significant increase in the cinnamic acid content, no changes in the contents of resveratrol, ferulic acid, sinapic acid, and epicatechin were observed. Furthermore, there was a significant positive correlation of TPC and TFC with 2,2-diphenyl-1-picrylhydrazyl scavenging capacity, 2,2-azino-bis (3-ethylbenzothiazoline-6-sulfonic acid) scavenging capacity, and ferric ion reducing antioxidant power in germinated peanut, but not with hydroxyl free radical scavenging capacity, in which the main monomer phenolic compounds were resveratrol, catechin, and quercetin. The research results indicate that microwave heating can effectively retain the phenolic substances and antioxidant activity in germinated peanuts, making it a more suitable ripening and processing method for germinated peanuts.

## 1. Introduction

Peanut (*Arachis hypogaea* L.), an annual herb of the genus Arachis, order Rosaceae, family Leguminosae, is known as “groundnut” or “everlasting fruit” among Asian populations [1]. Peanut is rich in fat, protein, and phenolic compounds and is popular with consumers as it can be eaten uncooked or cooked [2]. During peanut germination, large molecules, including fat, starch, and protein, are degraded into small molecules that can be easily absorbed by cells, and the contents of amino acids, sugars, vitamins, minerals, and antioxidants, such as the phenolic compound resveratrol, are highest during this period [3]. In recent years, the properties of peanut sprout have been investigated. Weng et al. [4] conducted a 35-day nutritional and toxicological evaluation of peanut sprout as a daily supplement in ICR mice, and no adverse effects were detected. Rao et al. [5] reported a significant reduction in the allergenic protein Ara h1 after peanut sprouting.

Sprouted peanuts are rich in resveratrol [6], which can promote health and prevent disease, such as helping to maintain a normal body weight, alleviating arthritic symptoms, and controlling diarrhea [7]. Moreover, consumption of sprouted peanut can reduce the risks of cancer, cardiovascular disease, and delay ageing [8,9]. It is evident that peanut sprouts have a variety of physiological effects, most of which are derived from the action of phenolic substances.

Phenols have important functions in plant growth and development. They are products of secondary metabolic processes and mainly converted from sugars through the pentose phosphate pathway, glycolytic pathway, mangiferous acid pathway, or benzene–propane pathway. In general, phenolics consist of monophenols such as benzenoids, and polyphenols such as flavonoids, stilbenoids, and coumuminoids [10]. Phenols have high antioxidant activity because they provide hydrogen ions and complex with metal ions, and sprouted peanut is a good source of antioxidants [11]. Adhikari et al. [7] observed that the contents of polyphenols and flavonoids in sprouted peanut were significantly higher than those in peanut seed. Moreover, Yu et al. [12] demonstrated that the resveratrol content in peanut sprout was about nine times higher than that in peanut seed. Limmongkon et al. [13] found that the total phenol content increased 7.6-fold and the total antioxidant capacity increased 9.5-fold after peanut germination.

Peanut sprout can be cooked in different ways. Cooking is accompanied by the release, degradation, and synthesis of phenolic compounds, resulting in qualitative, quantitative, and other changes; for example, different cooking methods have different effects on the phenolic compounds in peanut sprout [14,15]. Although the health benefits of peanut sprout have been studied in detail and the high resveratrol content has been demonstrated to underlie the beneficial effects, the impact of different cooking methods on phenol content and antioxidant activity has not been reported. In this study, we investigated the effects of five cooking methods, namely, steaming, boiling, roasting, microwave heating, and deep-frying, on the total phenol content, total flavonoid content, 2,2-diphenyl-1-picrylhydrazyl (DPPH) scavenging capacity, 2,2-azino-bis (3-ethylbenzothiazoline-6-sulfonic acid) (ABTS) scavenging capacity, ferric ion reducing antioxidant power (FRAP), and hydroxyl free radical scavenging capacity in peanut sprout. Using a correlation analysis and principal component analysis, the relationships of monomer phenolic compounds with antioxidant activity in peanut sprout were examined. Our findings provide a foundation for future research aimed at the industrial production of sprouted peanuts.

## 2. Results and Discussion

### 2.1. Effects of Different Cooking Methods on Total Phenol Content in Peanut Sprout

**Figure 1:**
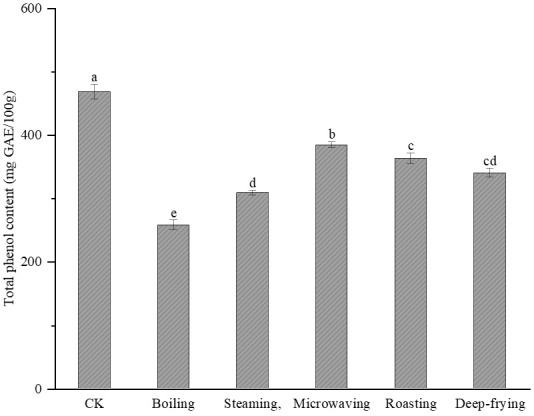
Effects of different cooking methods on total phenolic content of peanut sprout. Different lowercase letters indicate significant differences (*p* < 0.05). CK, control; GAE, gallic acid equivalents; DW, dry weight.

The TPC of peanut sprout after cooking is shown in Figure 1. The TPC decreased significantly (*p* < 0.05) after cooking by the different methods, with the TPCs of boiled, steamed, microwaved, roasted, and fried peanut sprout calculated as 258.02 ± 7.62 mg GAE/100 g, 309.45 ± 7.62 mg GAE/100 g, 384.24 ± 4.74 mg GAE/100 g, 363.47 ± 8.26 mg GAE/100 g, and 340.09 ± 6.78 mg GAE/100 g, respectively. Compared to uncooked peanut sprout, the TPC decreased by 33.97%, 44.87%, 17.95%, 22.44%, and 27.35%, respectively, where the highest retention of total phenols was demonstrated for microwave heating (82.05%) and the lowest retention of total phenols was demonstrated for boiling.

The mechanisms of phenol retention after cooking by the different methods are extremely complex. Heat reduces the activity of phenol oxidase and inhibits the oxidation and polymerization of phenols, and high temperature destroys cell walls and promotes phenol release from food. Furthermore, phenols are highly susceptible to transformation, polymerization, and degradation during heating, thus reducing their content; and the processing method, processing temperature, and processing time are important factors affecting their structure and content [16,17]. Phenols are poorly stabilized and easily degraded by heat, and they are lost in large quantities during heating [18]. At the same time, heat can disrupt the interactions between tannin and starch molecules, leading to the degradation of phenolic polymers and a reduction in tannin content, and the destruction of the tannin structure may be a factor in the reduction in the TPC [19]. del Pilar Ramírez–Anaya et al. [20] reported that potatoes, tomatoes, and squash had a significantly higher TPC when cooked with virgin olive oil than with water. This is because boiling causes many substances to polymerize and hydrolyze with heat and to be lost with water, resulting in a significant decrease in their content [21]. Giallourou et al. [22] demonstrated that the TPC of bean sprout was significantly lower after boiling, whereas microwaving retained most phenolic compounds. Yu et al. [23] suggested that the short time and high temperature of microwave heating were more conducive to the retention and release of phenolic compounds.

### 2.2. Effects of Different Cooking Methods on Total Flavonoid Content in Peanut Sprout

**Figure 2:**
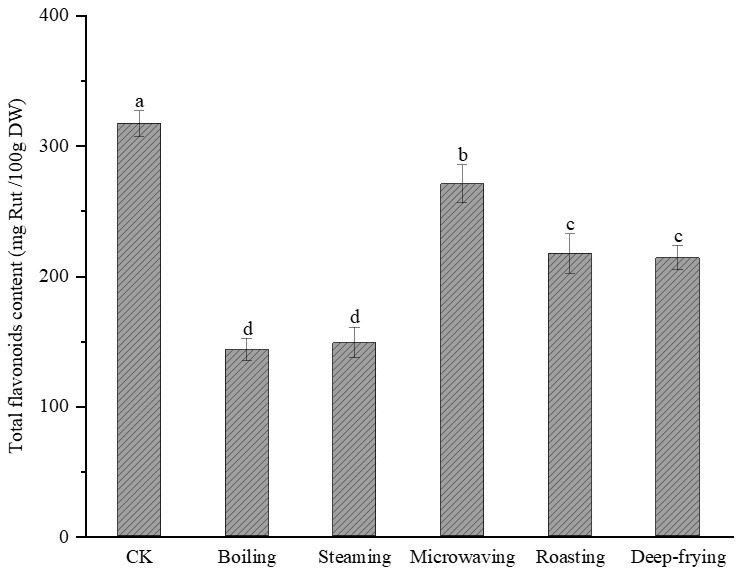
Effects of different cooking methods on total flavonoid content of peanut sprout. Different lowercase letters indicate significant differences (*p* < 0.05). CK, control; Rut, rutin; DW, dry weight.

The TFC in peanut sprout after cooking is shown in Figure 2. Similar to the change in TPC, there was a significant decrease (*p* < 0.05) in the TFC after cooking by the different methods. The TFCs of peanut sprout after boiling, steaming, microwave heating, roasting, and deep-frying were 142.91 ± 8.62 mg Rut/100 g, 148.43 ± 11.79 mg Rut/100 g, 270.3 ± 14.53 mg Rut/100 g, 216.83 ± 15.13 mg Rut/100 g, and 213.6 ± 9.22 mg Rut/100 g, with the highest retention of total flavonoids demonstrated for microwave heating (85.35%) and the lowest retention of total flavonoids demonstrated for boiling.

Flavonoids are unstable to a variety of factors such as light, pH, and temperature. The results of this study showed that flavonoids in sprouted peanut were more sensitive to temperature, especially after steaming and boiling, with more than 50% of flavonoids degraded. This is probably because during steaming and boiling, peanut sprout comes into contact with water. Furthermore, the heat area is large, and expansion and rupture lead to water loss as well as flavonoid degradation and loss, as is consistent with the results of previous studies [24,25]. Microwave heating is also destructive to cells and facilitates the release of flavonoids, although the short heating time and high heat transfer efficiency do not cause excessive evaporation of water from food, which facilitates the retention of flavonoids [26,27].

### 2.3. Effects of Different Cooking Methods on Antioxidant Activity in Peanut Sprout

**Figure 3:**
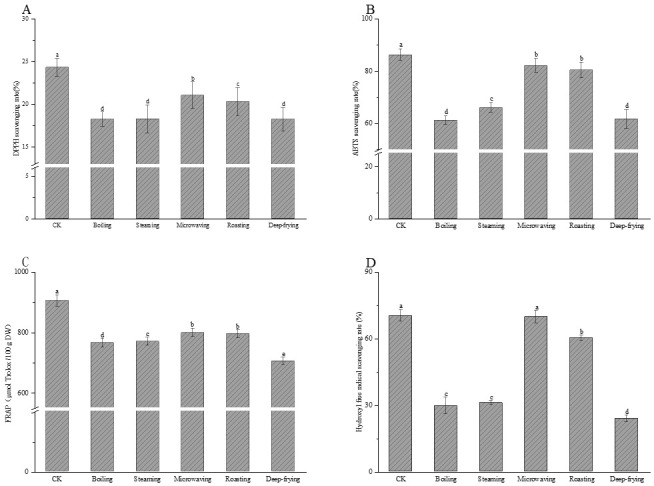
(**A**) 2, 2–diphenyl–1–picrylhydrazyl (DPPH) scavenging capacity; (**B**) 2, 2–azino–bis (3–ethylbenzothiazoline–6–sulfonic acid) (ABTS) scavenging capacity; (**C**) ferric ion reducing antioxidant power (FRAP); (**D**) hydroxyl free radical scavenging capacity. Different letters indicate significant differences (*p* < 0.05).

The effects of different cooking methods on DPPH scavenging capacity, ABTS scavenging capacity, FRAP, and the hydroxyl free radical scavenging capacity of peanut sprout are shown in Figure 3. As shown in Figure 3A, the DPPH scavenging rate in the microwaved group was 21.06%, which was significantly higher than that of the other four cooking methods (*p* < 0.05), and there was no significant difference between the boiled, steamed, and fried groups (*p* > 0.05), with the lowest DPPH scavenging rate in the boiled group. As shown in Figure 3B, the ABTS scavenging rate of peanut sprout in the microwave heating and roasting groups was 82.19%, which was significantly higher than that of the other three cooking methods (*p* < 0.05), and there was no significant difference between the boiling and deep-frying groups (*p* > 0.05). As shown in Figure 3C, the FRAP of the microwaved group was 798 ± 14.17 μmol Trolox/100 g, and that of the roasted group was 794 ± 13.78 μmol Trolox/100 g, both of which were significantly higher than that of the other three cooking methods (*p* < 0.05). There were significant differences between the boiled, steamed, and fried groups (*p* < 0.05), with the highest FRAP in the microwaved group and the lowest in the fried group. As shown in Figure 3D, the hydroxyl free radical scavenging rate in the microwaved group was 69.68%, which was not significantly lower than that of uncooked sprouted peanut (*p* > 0.05), and there was no significant difference between the boiled and steamed groups (*p* > 0.05), with the lowest hydroxyl free radical scavenging rate in the fried group. The results indicate that microwave heating is more suitable for peanut sprout cooking, and the cooking method can retain most antioxidants.

The changes in the four antioxidant capacities (DPPH scavenging capacity, ABTS scavenging capacity, FRAP, and hydroxyl free radical scavenging capacity) of peanut sprout that was cooked by different methods were generally consistent with the changes in the TPC and TFC. These results indicate that the antioxidant capacity of cooked peanut sprout was related to the processing method as well as the retention of phenols and flavonoids. Boukhanouf et al. [28] concluded that the antioxidant capacity generally varies with the TPC during processing. Liu et al. [29] reported that heat treatment reduces the antioxidant activity of sprouted brown rice and that the trend is directly related to changes in the TPC. Ti et al. [30] arrived at similar conclusions after evaluating the antioxidant activities of different varieties of rice; they reported that a single antioxidant activity assay cannot provide a comprehensive and effective evaluation of the antioxidants present in a complex food system. Therefore, a combination of methods is needed to evaluate antioxidant activity. Amin et al. [31] concluded that there was no positive correlation between antioxidant activity and TPC, which was mainly due to the different content of each component and the different stability of each monomer phenolic compound in the various food types. Phenol structure also affects antioxidant activity. The antioxidant activity of monomeric phenol compounds is related to the size of the hydroxide bond dissociation energy, the stability of bound free radicals, and the formation of new compounds with strong antioxidant activity. Different cooking methods affect the activity of monomeric phenols to different degrees, and different monomeric phenols have different selectivities for scavenging free radicals [32]. Compared to conventional cooking methods, microwave heating is a cold heat source, not a direct heat transfer source; thus, it has less impact on heat-sensitive monomeric phenol compounds [33,34].

### 2.4. Effects of Different Cooking Methods on Monomer Phenolic Compounds in Peanut Sprout

The monomer phenolic content of peanut sprout after cooking is shown in Table 1. The monomer phenolic content of peanut sprout changed after cooking, which was due to the different thermal sensitivities of the different monomer phenolic compounds and the effect of the heating method on the compounds’ structure. The content of each monomer phenolic compound decreased significantly (*p* < 0.05) after boiling compared to steaming and deep-frying, as is consistent with previous changes in the TPC. After microwave heating, there was no significant change (*p* > 0.05) in the contents of resveratrol, ferulic acid, sinapic acid, and epicatechin, but a significant increase (*p* < 0.05) in the content of cinnamic acid, and a significant decrease (*p* < 0.05) in the contents of all other monomeric phenols. Resveratrol is the most representative functional substance in peanut sprout, and it is important to retain its activity during cooking. After roasting, the epicatechin content did not change significantly (*p* > 0.05), the cinnamic acid content increased significantly (*p* < 0.05), and the contents of all other monomeric phenols decreased. The effects of the different cooking methods on the monomeric phenols were different, and even when the same cooking method was used, the changes in monomer phenols were different; the effects were related to the type, variety, genotype, and maturity of the food, but also closely related to the cooking method and processing conditions [35].

### 2.5. Correlation Analysis of Phenolic Compounds and Antioxidant Activities of Peanut Sprout

**Figure 4:**
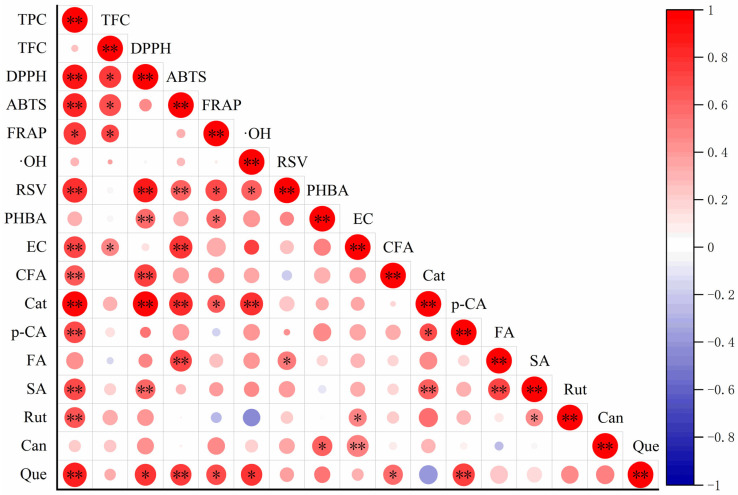
Pearson correlation analysis of monomer phenols and antioxidant activity. * indicates a significant difference at *p* < 0.05; ** indicates a significant difference at *p* < 0.01. TPC, total phenol content; TFC, total flavonoid content; DPPH, 2,2-diphenyl-1-picrylhydrazyl scavenging capacity; ABTS, 2,2-azino-bis (3-ethylbenzothiazoline-6-sulfonic acid) scavenging capacity; FRAP, ferric ion reducing antioxidant power; ·OH, hydroxyl free radical scavenging capacity; RSV, resveratrol; PHBA, p–hydroxybenzoic; EC, epicatechin; CFA, caffeic acid; Cat, catechin; p–CA, p–coumaric acid; FA, ferulic acid; SA, sinapic acid; Rut, rutin; Can, cinnamic acid; Que, quercetin.

To investigate the relationship between the phenolic content and antioxidant activity of peanut sprout, Pearson’s correlation coefficient was used, and the results are shown in Figure 4. The results of the correlation analysis showed that there was a highly significant (*p* < 0.01) correlation between TPC, DPPH scavenging capacity, and ABTS scavenging capacity, whereas there was a significant (*p* < 0.05) correlation between TFC, DPPH scavenging capacity, and ABTS scavenging capacity. There was also a significant (*p* < 0.05) correlation between TPC, TFC, and FRAP, which proves that phenols and flavonoids are the main compounds with antioxidant properties in peanut sprout. TPC, TFC, and hydroxyl free radical scavenging activity were positively correlated, but the correlation was not significant (*p* > 0.05), which may be due to the presence of other antioxidants in peanut sprout with stronger activity in the hydroxyl free radical scavenging system. All 11 monomeric phenols were positively correlated with DPPH scavenging capacity, with highly significant (*p* < 0.01) correlations between DPPH scavenging capacity, resveratrol, p–hydroxybenzoic, caffeic acid, catechin, and sinapic acid. There was a significant (*p* < 0.05) correlation between DPPH scavenging capacity and quercetin; a non-significant correlation (*p* > 0.05) between ABTS scavenging capacity, rutin, and cinnamic acid; and a highly significant (*p* < 0.01) correlation between ABTS, resveratrol, epicatechin, catechin, ferulic acid, and quercetin. There was a significant (*p* < 0.05) correlation between FRAP, resveratrol, p–hydroxybenzoic, catechin, and quercetin and a negative correlation between FRAP, p–coumaric acid, and rutin. There was a highly significant (*p* < 0.01) correlation between catechin and hydroxyl free radical scavenging activity; a significant (*p* < 0.05) correlation between resveratrol, quercetin, and hydroxyl free radical scavenging activity; and a negative correlation between caffeic acid and hydroxyl free radical scavenging activity. These results indicate that the main antioxidants in peanut sprout are resveratrol, catechin, and quercetin, and that the correlations between different monomeric phenols and different antioxidant properties are variable.

### 2.6. Principal Component Analysis of Cooking Methods, Phenolic Compounds, and Antioxidant Activities

**Figure 5:**
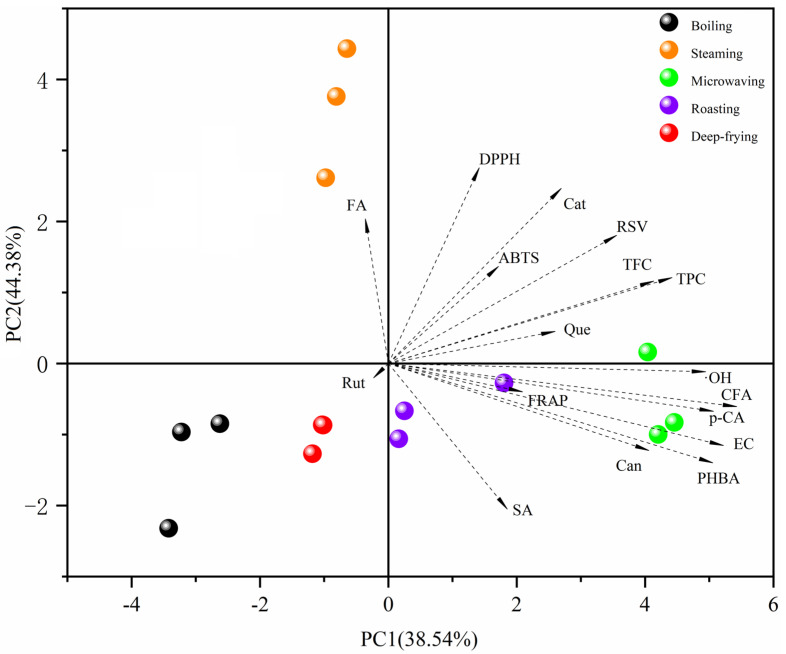
Principal component analysis of cooking methods, phenolic compounds, and antioxidant activities.

To further analyze the effects of different cooking methods on the phenolic compounds and antioxidant activities of peanut sprout, a principal component analysis was conducted on the TPC, TFC, 11 phenolic compounds, and four antioxidant activities in peanut sprout. As shown in Figure 5, the contribution of PC1 was 38.54%, the contribution of PC2 was 44.38%, and the contribution of both principal components was 82.92%. The five cooking methods were clustered and distinguished into five different groups, indicating significant differences in the effects of different cooking methods on the phenolic content and antioxidant activity of peanut sprout.

In PC1, most monomeric phenols, except ferulic acid and rutin, were on the positive semi-axis with the four antioxidants of sprouted peanuts. In PC2, most monomeric phenols were not on the same semi-axis with the four antioxidants of sprouted peanut, indicating that there were differences in the antioxidant capacities of the different monomeric phenols; and those in the same quadrant with the antioxidant capacities were TPC, TFC, resveratrol, catechin, and quercetin, revealing these substances are the main antioxidants in peanut sprout. These results are consistent with those of the correlation analysis.

## 3. Materials and Methods

### 3.1. Peanut Seeds and Chemicals

The peanut cultivar Fuhua 22 was used, which is from Liaoning Province, China. All peanut seeds were obtained from the Liaoning Academy of Agricultural Sciences in mid-October 2021. All chemicals and solvents were of analytic or chromatographic grade and purchased from Sinopharm Chemical Reagent Co., Ltd. (Beijing, China) or Solarbio (Beijing, China).

### 3.2. Peanut Germination

Peanut (1000 g) was sterilized in 1% (*w/v*) NaClO solution for 13 min, rinsed with deionized water, and soaked in 2 L of distilled water for 4 h. Peanut kernels were placed on a ceramic tray and germinated in a growth chamber (BD–ZGX–400G–4P, Nanjing Beidi Instrument Co., Ltd., Nanjing, China) at 26 °C for 24 h in the dark. Peanut kernels were transplanted to a sterilized sprouting tray, distilled water was added to the bottom of the tray, and the tray was incubated in a growth chamber at a temperature of 28 °C and a relative humidity of 80%. The water was changed every 24 h. Peanut was harvested after 6 d of germination. The sprouted peanut was divided into two batches: the first batch was used for cooking and the second batch was freeze-dried (FreeZone2.5 + CentriVap, Labconco Corp., Kansas City, MO, USA). The sprouted peanut was freeze–dried, crushed, passed through a 60-mesh screen to obtain peanut powder, and stored in a freezer (MDF–U54V, Sanyo, Japan) at −80 °C.

### 3.3. Preparation of Peanut Sprout Products by Different Cooking Methods

Peanut sprout was boiled and steamed, as described by Chukwumah et al. [36], with some modifications. For boiling, 100 g of the peanut sprout was added to 1 L of distilled water, boiled for 10 min, and cooled to room temperature. For steaming, water was boiled. The peanut sprout (100 g) was added to a steamer rack, steamed for 10 min, and cooled to room temperature. Then, it was microwaved as described by Raigar et al. [37]. Following this, the peanut sprout was added to a ceramic dish, microwaved at 500–W power for 3 min, and cooled to room temperature. It was then roasted, as described by Ferreira et al. [38], with some modifications. The oven was heated to 150 °C. The peanut sprout was added to a baking tray, baked for 10 min, and cooled to room temperature. Afterwards, it was deep-fried, as described by Srichamnong et al. [39]. Soybean oil (500 mL) was heated to 150 °C. The peanut sprout was deep-fried for 5 min, drained, and cooled to room temperature for later use. The above samples were freeze-dried, crushed, and passed through a 60-mesh screen to obtain cooked peanut sprout powder.

### 3.4. Preparation of Peanut Sprout Extract

Cooked peanut sprout powder (1 g) was added to 30 mL of 70% (*v*/*v*) ethanol solution in a 50 mL centrifuge tube, extracted in an ultrasonic bath (KQ–300VDE, Kunshan Ultrasonic Instrument Co., Ltd., Kunshan City, China) at 47 °C for 30 min, and centrifuged at 2500 g (TGL20M, Hunan Xiangyi Laboratory Instrument Development Co., Ltd., Changsha, China) for 15 min. The supernatant was collected.

### 3.5. Determination of Total Phenol Content

**Figure 6:**
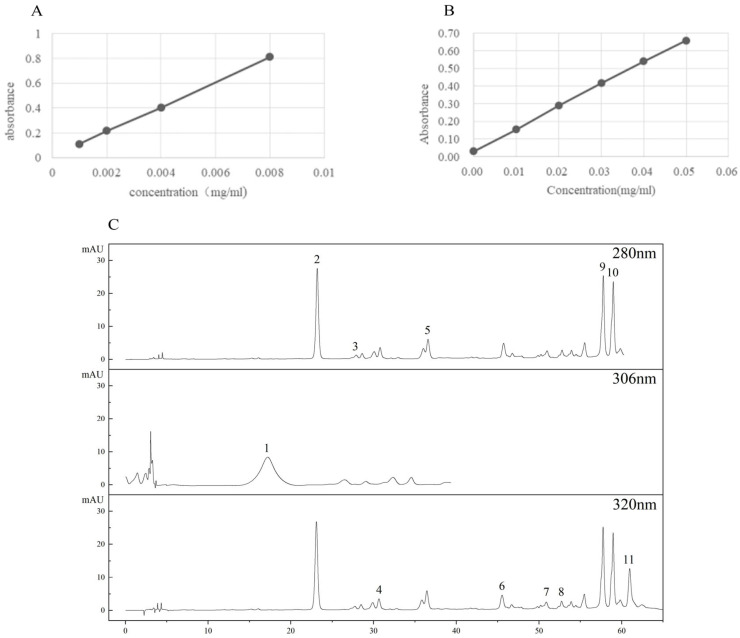
(**A**) Calibration curves for gallic acid; (**B**) calibration curves for rutin; (**C**) the HPLC profile of phenolic compounds. 1, resveratrol; 2, p–Hydroxybenzoic acid; 3, epicatechin; 4, caffeic acid; 5, catechin; 6, p-Coumaric acid; 7, ferulic acid; 8, sinapic acid; 9, rutin; 10, cinnamic acid; 11, quercetin.

The total phenol content (TPC) was determined, as described by Kim et al. [40], with some modifications. Peanut sprout extract (0.5 mL) was added to 1 mL of forint phenol color developer and mixed. Next, 1 mL of 7.5% (*w/v*) 7.95 M Na_2_CO_3_ solution was added, the volume was adjusted to 10 mL with distilled water, and the sample was mixed and incubated at room temperature for 2 h in the dark. The absorbance at 765 nm was measured with a UV–Vis spectrophotometer (UV–5100, Shanghai Metash Instruments Co., Ltd., Shanghai, China). A reagent blank served as a reference, and a standard curve was drawn using gallic acid equivalents (GAE) as the standard (Figure 6A). The TPC content is calculated based on the mass of GAE per 100 g of sample dry basis, and the result is expressed as mg GAE/100 g.

### 3.6. Determination of Total Flavonoid Content

The total flavonoid content (TFC) was determined, as described by Dewanto et al. [41], with some modifications. Peanut sprout extract (0.5 mL) was added to 0.5 mL of 5% (*w/v*) 4.25 M NaNO₂ solution, mixed, and incubated at room temperature for 6 min. Next, 0.5 mL of 10% (*w/v*) 21.3 M Al(NO₃)₃ solution was added, mixed, and incubated at room temperature for 6 min, after which 4 mL of 1 M NaOH solution was added and diluted to 10 mL with 30M ethanol. The absorbance at 510 nm was measured with a UV–Vis spectrophotometer (UV–5100, Shanghai Metash Instruments Co., Ltd., Shanghai, China). Using reagent blank as reference and rutin (Rut) as standard, a standard curve was drawn (Figure 6B). TFC is calculated as the mass of Rut per 100 g of sample dry basis, and the result is expressed as mg Rut/100 g.

### 3.7. Determination of Antioxidant Activity

The antioxidant properties of peanut sprout were evaluated by determining the DPPH scavenging capacity, ABTS scavenging capacity, FRAP, and hydroxyl free radical scavenging capacity, followed by comparative analyses.

#### 3.7.1. Determination of DPPH Scavenging Capacity

Peanut sprout extract (0.5 mL) was added to 2.0 mL of 0.1 mM DPPH reagent, mixed, and incubated at room temperature for 30 min in the dark. The absorbance at 515 nm was measured. Methanol (0.5 mL) and DPPH reagent (2.0 mL) served as a control. The DPPH scavenging capacity was calculated as follows:

(1) $$\mathrm{Clearance}\;\mathrm{rate}\;(\%)=\frac{A_{0}-A_{1}}{A_{0}}\times 100\%$$

*A*_0_: sample absorbance;

*A*_1_: control absorbance.

#### 3.7.2. Determination of ABTS Scavenging Capacity

A master mix was prepared by combining 7 mM of ABTS solution with 2.45 mM of K_2_S_2_O_8_ at a ratio of 1:1 (*v*/*v*) and incubating at room temperature for 12 h in the dark. Peanut sprout extract (0.3 mL) was added to 2.7 mL of ABTS working solution, mixed, and incubated at 30 °C for 6 min. The absorbance at 405 nm was measured. Methanol (0.3 mL) combined with ABTS working solution (2.7 mL) served as a control. The ABTS scavenging capacity was calculated as follows:

(2) $$\mathrm{Scavenging}\;\mathrm{rate}\;(\%)=\frac{A_{0}-A_{1}}{A_{0}}\times 100\%$$

*A*_0_: sample absorbance;

*A*_1_: control absorbance.

#### 3.7.3. Determination of FRAP

The determination of FRAP was carried out, as described by Ti et al. [30], with some modifications. 2,4,6–tripyridin–2–yl–1,3,5–triazine solution was diluted with 40 mM of HCl and combined with 20 mM of FeCl₃ solution at a ratio of 10:1:1 (*v*/*v*/*v*) at 37 °C. Peanut sprout extract (0.075 mL) was added to 0.85mL of FRAP working solution, mixed, and incubated at 25 °C for 10 min. The absorbance at 590 nm was measured using a UV spectrophotometer. To prepare the standard curve, Trolox standard solutions with mass concentrations of 0.16 M, 0.32 M, 0.48 M, 0.64 M, and 0.8 M were prepared, and the absorbance values were determined, with the molar mass of Trolox solution (nmol) plotted as the horizontal coordinate. The results of FRAP were expressed as μmol Trolox equivalent per 100 g of sample dry weight (μmol Trolox/100 g).

#### 3.7.4. Determination of Hydroxyl Free Radical Scavenging Capacity

The BC1320 Hydroxyl Free Radical Scavenging Capacity Kit (Solarbio Science & Technology Co., Ltd., Beijing, China) was used to determine the hydroxyl free radical scavenging capacity, according to the manufacturer’s instructions.

### 3.8. Determination of Phenolic Composition

The phenolic composition was determined, as described by Liu et al. [42], with some modifications. The 1260 High Performance Liquid Chromatography System (Agilent Technologies, Palo Alto, CA, USA) with a diode array detector and an Eclipse Plus C18 Column (4.6 × 250 mm, 5 μm, Agilent Technologies) was used. The mobile phase was 1% (*v*/*v*) glacial acetic acid (A) and methanol (B) at a flow rate of 0.8 mL/min at 35 °C. The detection wavelengths were 306 nm, 280 nm, and 320 nm. Monomeric phenols were analyzed qualitatively and quantitatively using a standard method, according to the retention time and peak area of each standard (Table 2). The HPLC spectrum of phenolic compounds in germinated peanuts is shown in Figure 6C. The content of monomeric phenols was expressed as μg per g of sample dry weight (Table 1).

### 3.9. Data Analysis

Data were processed and plotted using SPSS 17.0 (SPSS Inc., Chicago, IL, USA) and Origin 2021 (OriginLab, Northampton, MA, USA) software packages, respectively. All experiments were repeated three independent times, and all results were expressed as mean ± standard deviation.

## 4. Conclusions

The phenol content and antioxidant activity of sprouted peanut were significantly affected by different cooking methods. The TPC and TFC decreased significantly after boiling, steaming, microwaving, roasting, and deep-frying, with the highest retention of both caused by microwave heating and the lowest retention caused by boiling. The antioxidant properties of sprouted peanut were reduced to varying degrees after cooking, and similar to the TPC and TFC, microwaving retained maximum antioxidant activity. The monomer phenolic content of sprouted peanut changed after cooking, and there were no significant changes in the contents of resveratrol, ferulic acid, sinapic acid, and epicatechin; further, a significant increase was observed in the cinnamic acid content after microwave heating. The correlation between 11 monomeric phenols and antioxidant activity was variable, with resveratrol, catechin, and quercetin functioning as the main antioxidants. Microwave heating is an effective way to retain the phenol content and antioxidant activity of sprouted peanut.

## 5. Limitations

Chinese cooking is highly complicated and most of it does not follow one cooking method. We only analyzed the effects of a single cooking method on phenolic substances and antioxidant activities in peanut sprout; the effects of combined cooking methods require further research.

## Figures and Tables

**Table 1:** Contents of phenolic compounds in peanut sprout by different cooking methods.

	CK (μg/g DW)	Boiling (μg/g DW)	Steaming (μg/g DW)	Microwave Heating (μg/g DW)	Roasting (μg/g DW)	Deep-Frying (μg/g DW)
Resveratrol	25.83 ± 1.89 ^a^	14.26 ± 1.37 ^b^	9.95 ± 1.88 ^c^	24.68 ± 0.42 ^a^	11.16 ± 3.38 ^c^	9.13 ± 1.36 ^c^
p–Hydroxybenzoic acid	595.73 ± 28.90 ^a^	470.05 ± 63.82 ^b^	305.06 ± 10.41 ^d^	478.34 ± 33.81 ^b^	432.77 ± 6.73 ^bc^	403.26 ± 25.88 ^c^
Epicatechin	168.64 ± 2.42 ^ab^	134.57 ± 12.23 ^cd^	107.37 ± 16.00 ^d^	173.73 ± 26.54 ^a^	149.73 ± 10.73 ^abc^	139.84 ± 15.38 ^bc^
Caffeic acid	1.60 ± 0.20 ^a^	0.72 ± 0.08 ^b^	0.35 ± 0.04 ^c^	0.45 ± 0.09 ^c^	0.43 ± 0.02 ^c^	0.65 ± 0.11 ^b^
Catechin	70.97 ± 2.60 ^a^	1.10 ± 1.05 ^f^	4.44 ± 1.89 ^e^	34.27 ± 2.37 ^b^	23.31 ± 0.89 ^c^	16.15 ± 0.83 ^d^
p–Coumaric acid	144.90 ± 8.33 ^a^	97.54 ± 3.56 ^c^	82.01 ± 5.87 ^d^	111.06 ± 3.20 ^b^	76.56 ± 1.11 ^d^	107.60 ± 11.28 ^bc^
Ferulic acid	27.17 ± 3.67 ^a^	18.59 ± 2.28 ^b^	18.55 ± 2.15 ^b^	27.14 ± 1.24 ^a^	21.45 ± 1.29 ^b^	8.89 ± 1.54 ^c^
Sinapic acid	4.24 ± 0.12 ^a^	3.28 ± 0.16 ^c^	4.03 ± 0.10 ^b^	4.18 ± 0.36 ^a^	3.83 ± 0.08 ^b^	3.75 ± 0.41 ^b^
Rutin	67.33 ± 1.28 ^a^	52.39 ± 1.17 ^c^	51.36 ± 1.04 ^c^	62.80 ± 1.58 ^b^	61.83 ± 2.41 ^b^	62.15 ± 0.59 ^b^
Cinnamic acid	90.26 ± 5.57 ^b^	95.49 ± 2.76 ^b^	68.86 ± 3.67 ^c^	102.20 ± 5.14 ^a^	100.25 ± 2.61 ^a^	69.69 ± 0.85 ^c^
Quercetin	6.20 ± 0.52 ^a^	1.71 ± 0.17 ^d^	1.66 ± 0.03 ^d^	5.70 ± 0.10 ^b^	2.68 ± 0.04 ^c^	1.87 ± 0.13 ^d^

Note: Different lowercase letters indicate statistically significant differences among different cooking methods (*p* < 0.05). CK, control; DW, dry weight.

**Table 2:** Standard curves for phenolic compounds.

Phenolic Compound	Equation	R^2^	Retention Time/min
Resveratrol	y = 44.725x + 14.001	0.9996	15.302
p–Hydroxybenzoic acid	y = 18.062x + 5.9625	0.9996	23.787
Epicatechin	y = 3.7768x + 4.2018	0.9998	25.711
Caffeic acid	y = 726.29x − 1.5116	0.9996	32.984
Catechin	y = 49.1x + 34.222	0.9995	39.128
p–Coumaric acid	y = 50.49x − 36.573	0.9996	45.698
Ferulic acid	y = 41.891x + 33.402	0.9999	50.978
Sinapic acid	y = 692.7x − 20.507	0.9996	52.904
Rutin	y = 103.12x − 15.667	0.9999	56.009
Cinnamic acid	y = 60.505x − 13.032	0.9999	57.819
Quercetin	y = 61.857x − 1.9518	0.9997	61.47

## Data Availability

The data presented in this study are available on request from the corresponding author.
